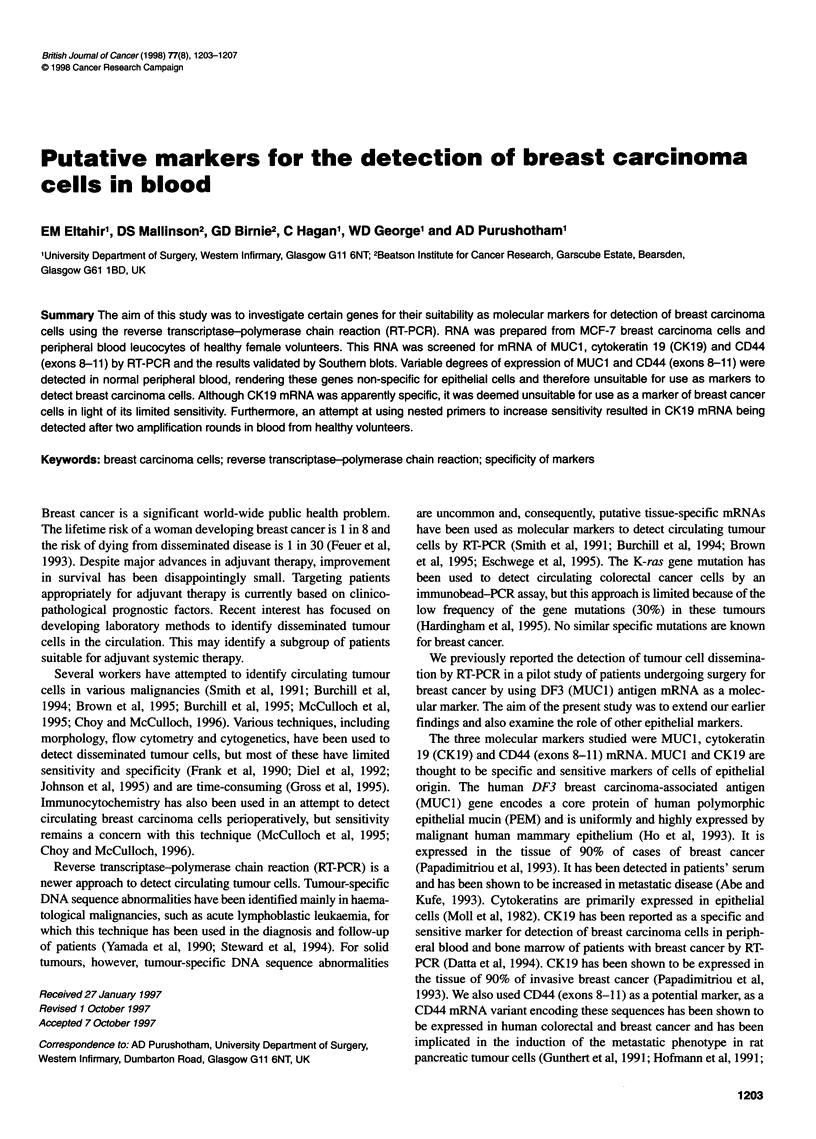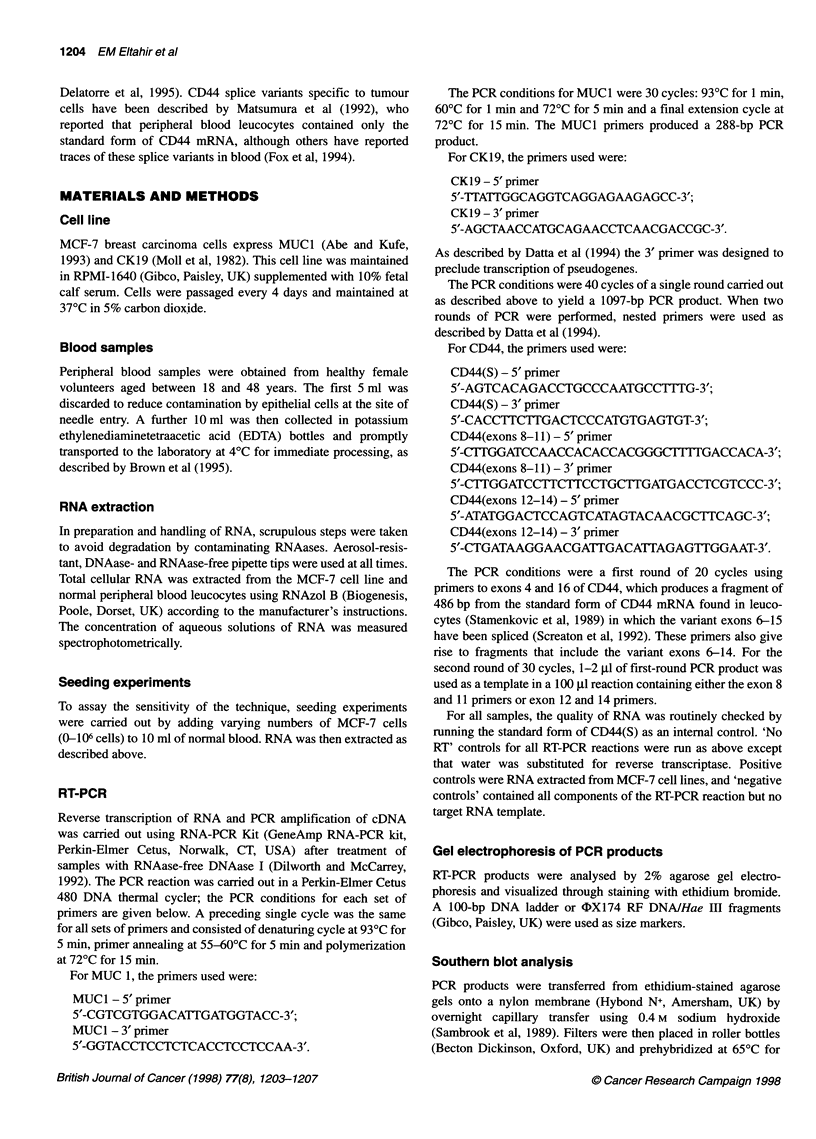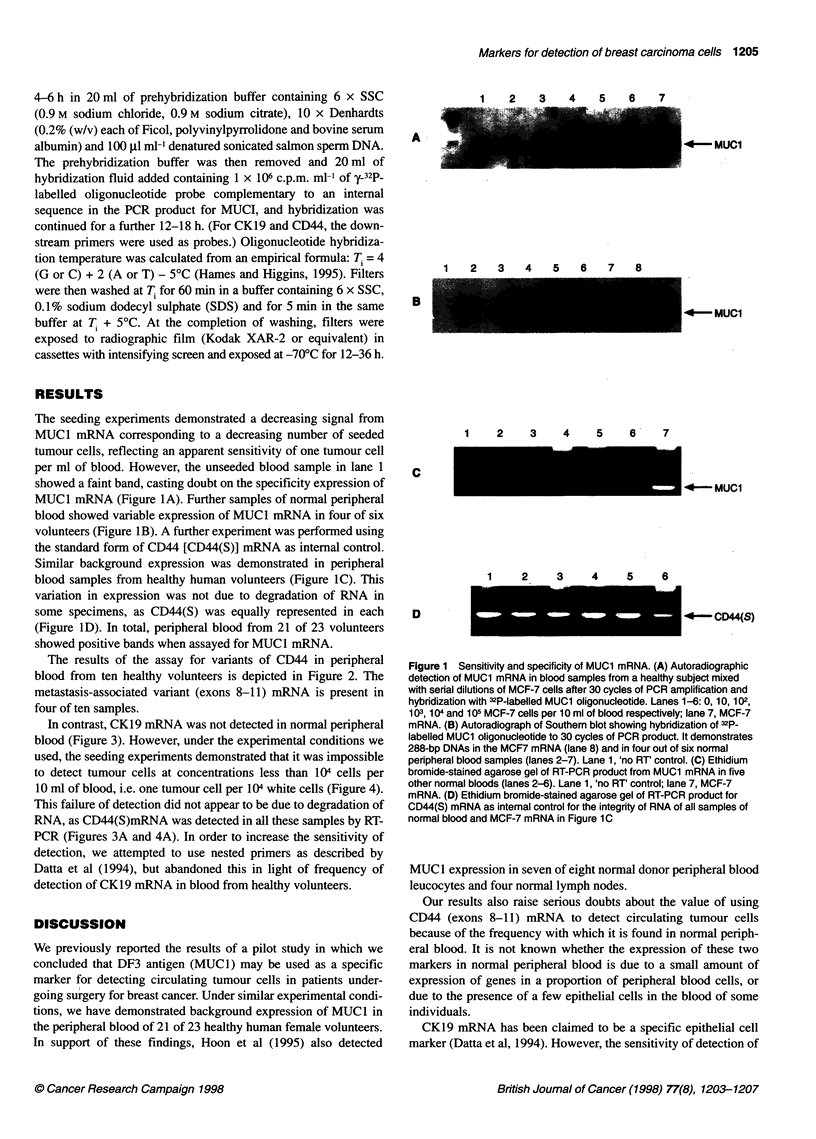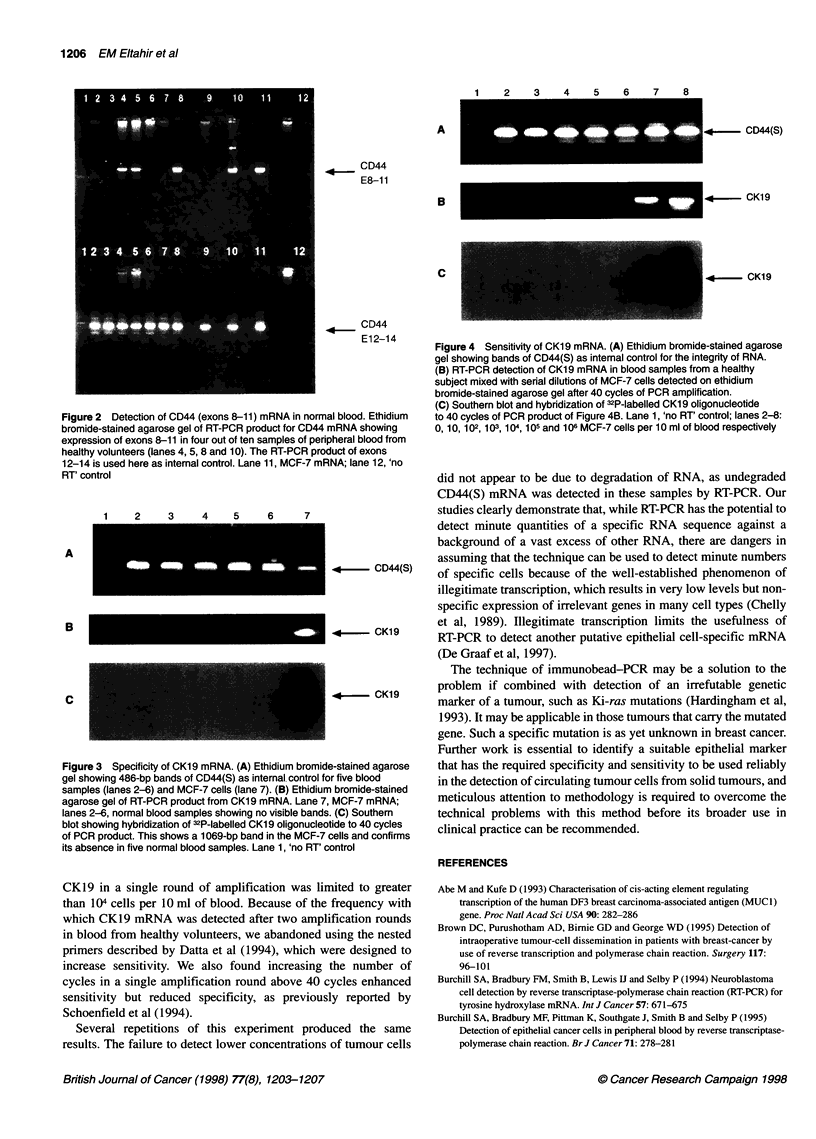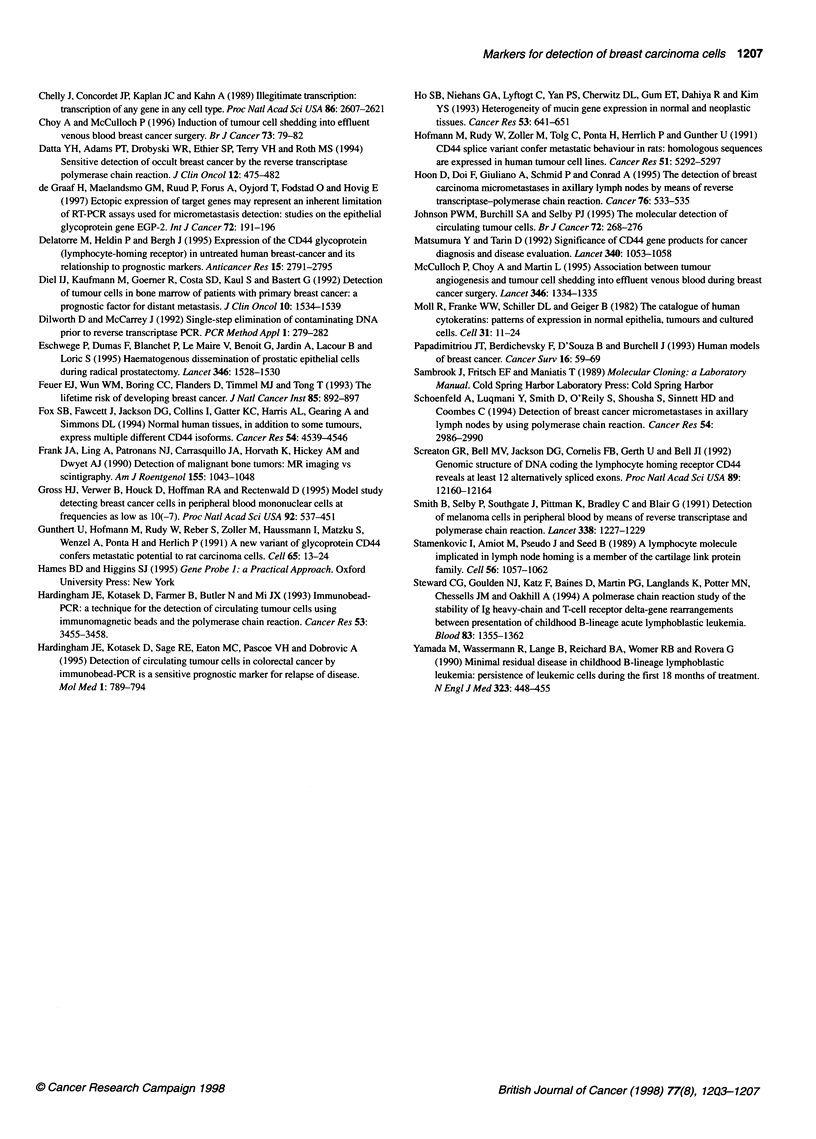# Putative markers for the detection of breast carcinoma cells in blood.

**DOI:** 10.1038/bjc.1998.203

**Published:** 1998-04

**Authors:** E. M. Eltahir, D. S. Mallinson, G. D. Birnie, C. Hagan, W. D. George, A. D. Purushotham

**Affiliations:** University Department of Surgery, Western Infirmary, Glasgow, UK.

## Abstract

**Images:**


British Journal of Cancer (1998) 77(8), 1203–1207
© 1998 Cancer Research Campaign

### Putative markers for the detection of breast carcinoma cells in blood

**EM Eltahir[1], DS Mallinson[2], GD Birnie[2], C Hagan[1], WD George[1] and AD Purushotham[1]**

[1]University Department of Surgery, Western Infirmary, Glasgow G11 6NT; [2]Beatson Institute for Cancer Research, Garscube Estate, Bearsden, Glasgow G61 1BD, UK

**Summary** The aim of this study was to investigate certain genes for their suitability as molecular markers for detection of breast carcinoma cells using the reverse transcriptase–polymerase chain reaction (RT-PCR). RNA was prepared from MCF-7 breast carcinoma cells and peripheral blood leucocytes of healthy female volunteers. This RNA was screened for mRNA of MUC1, cytokeratin 19 (CK19) and CD44 (exons 8–11) by RT-PCR and the results validated by Southern blots. Variable degrees of expression of MUC1 and CD44 (exons 8–11) were detected in normal peripheral blood, rendering these genes non-specific for epithelial cells and therefore unsuitable for use as markers to detect breast carcinoma cells. Although CK19 mRNA was apparently specific, it was deemed unsuitable for use as a marker of breast cancer cells in light of its limited sensitivity. Furthermore, an attempt at using nested primers to increase sensitivity resulted in CK19 mRNA being detected after two amplification rounds in blood from healthy volunteers.

**Keywords:** breast carcinoma cells; reverse transcriptase–polymerase chain reaction; specificity of markers

Breast cancer is a significant world-wide public health problem. The lifetime risk of a woman developing breast cancer is 1 in 8 and the risk of dying from disseminated disease is 1 in 30 (Feuer et al, 1993). Despite major advances in adjuvant therapy, improvement in survival has been disappointingly small. Targeting patients appropriately for adjuvant therapy is currently based on clinicopathological prognostic factors. Recent interest has focused on developing laboratory methods to identify disseminated tumour cells in the circulation. This may identify a subgroup of patients suitable for adjuvant systemic therapy.

Several workers have attempted to identify circulating tumour cells in various malignancies (Smith et al, 1991; Burchill et al, 1994; Brown et al, 1995; Burchill et al, 1995; McCulloch et al, 1995; Choy and McCulloch, 1996). Various techniques, including morphology, flow cytometry and cytogenetics, have been used to detect disseminated tumour cells, but most of these have limited sensitivity and specificity (Frank et al, 1990; Diel et al, 1992; Johnson et al, 1995) and are time-consuming (Gross et al, 1995). Immunocytochemistry has also been used in an attempt to detect circulating breast carcinoma cells perioperatively, but sensitivity remains a concern with this technique (McCulloch et al, 1995; Choy and McCulloch, 1996).

Reverse transcriptase–polymerase chain reaction (RT-PCR) is a newer approach to detect circulating tumour cells. Tumour-specific DNA sequence abnormalities have been identified mainly in haematological malignancies, such as acute lymphoblastic leukaemia, for which this technique has been used in the diagnosis and follow-up of patients (Yamada et al, 1990; Steward et al, 1994). For solid tumours, however, tumour-specific DNA sequence abnormalities are uncommon and, consequently, putative tissue-specific mRNAs have been used as molecular markers to detect circulating tumour cells by RT-PCR (Smith et al, 1991; Burchill et al, 1994; Brown et al, 1995; Eschwege et al, 1995). The K-*ras* gene mutation has been used to detect circulating colorectal cancer cells by an immunobead–PCR assay, but this approach is limited because of the low frequency of the gene mutations (30%) in these tumours (Hardingham et al, 1995). No similar specific mutations are known for breast cancer.

We previously reported the detection of tumour cell dissemination by RT-PCR in a pilot study of patients undergoing surgery for breast cancer by using DF3 (MUC1) antigen mRNA as a molecular marker. The aim of the present study was to extend our earlier findings and also examine the role of other epithelial markers.

The three molecular markers studied were MUC1, cytokeratin 19 (CK19) and CD44 (exons 8–11) mRNA. MUC1 and CK19 are thought to be specific and sensitive markers of cells of epithelial origin. The human *DF3* breast carcinoma-associated antigen (MUC1) gene encodes a core protein of human polymorphic epithelial mucin (PEM) and is uniformly and highly expressed by malignant human mammary epithelium (Ho et al, 1993). It is expressed in the tissue of 90% of cases of breast cancer (Papadimitriou et al, 1993). It has been detected in patients' serum and has been shown to be increased in metastatic disease (Abe and Kufe, 1993). Cytokeratins are primarily expressed in epithelial cells (Moll et al, 1982). CK19 has been reported as a specific and sensitive marker for detection of breast carcinoma cells in peripheral blood and bone marrow of patients with breast cancer by RT-PCR (Datta et al, 1994). CK19 has been shown to be expressed in the tissue of 90% of invasive breast cancer (Papadimitriou et al, 1993). We also used CD44 (exons 8–11) as a potential marker, as a CD44 mRNA variant encoding these sequences has been shown to be expressed in human colorectal and breast cancer and has been implicated in the induction of the metastatic phenotype in rat pancreatic tumour cells (Gunthert et al, 1991; Hofmann et al, 1991;

*Received 27 January 1997*
*Revised 1 October 1997*
*Accepted 7 October 1997*

*Correspondence to:* AD Purushotham, University Department of Surgery, Western Infirmary, Dumbarton Road, Glasgow G11 6NT, UK

Delatorre et al, 1995). CD44 splice variants specific to tumour cells have been described by Matsumura et al (1992), who reported that peripheral blood leucocytes contained only the standard form of CD44 mRNA, although others have reported traces of these splice variants in blood (Fox et al, 1994).

### MATERIALS AND METHODS

### Cell line

MCF-7 breast carcinoma cells express MUC1 (Abe and Kufe, 1993) and CK19 (Moll et al, 1982). This cell line was maintained in RPMI-1640 (Gibco, Paisley, UK) supplemented with 10% fetal calf serum. Cells were passaged every 4 days and maintained at 37°C in 5% carbon dioxide.

### Blood samples

Peripheral blood samples were obtained from healthy female volunteers aged between 18 and 48 years. The first 5 ml was discarded to reduce contamination by epithelial cells at the site of needle entry. A further 10 ml was then collected in potassium ethylenediaminetetraacetic acid (EDTA) bottles and promptly transported to the laboratory at 4°C for immediate processing, as described by Brown et al (1995).

### RNA extraction

In preparation and handling of RNA, scrupulous steps were taken to avoid degradation by contaminating RNAases. Aerosol-resistant, DNAase- and RNAase-free pipette tips were used at all times. Total cellular RNA was extracted from the MCF-7 cell line and normal peripheral blood leucocytes using RNAzol B (Biogenesis, Poole, Dorset, UK) according to the manufacturer's instructions. The concentration of aqueous solutions of RNA was measured spectrophotometrically.

### Seeding experiments

To assay the sensitivity of the technique, seeding experiments were carried out by adding varying numbers of MCF-7 cells ($0–10^6$ cells) to 10 ml of normal blood. RNA was then extracted as described above.

### RT-PCR

Reverse transcription of RNA and PCR amplification of cDNA was carried out using RNA-PCR Kit (GeneAmp RNA-PCR kit, Perkin-Elmer Cetus, Norwalk, CT, USA) after treatment of samples with RNAase-free DNAase I (Dilworth and McCarrey, 1992). The PCR reaction was carried out in a Perkin-Elmer Cetus 480 DNA thermal cycler; the PCR conditions for each set of primers are given below. A preceding single cycle was the same for all sets of primers and consisted of denaturing cycle at 93°C for 5 min, primer annealing at 55–60°C for 5 min and polymerization at 72°C for 15 min.

For MUC 1, the primers used were:

MUC1 – 5′ primer
5′-CGTCGTGGACATTGATGGTACC-3′;
MUC1 – 3′ primer
5′-GGTACCTCCTCTCACCTCCTCCAA-3′.

The PCR conditions for MUC1 were 30 cycles: 93°C for 1 min, 60°C for 1 min and 72°C for 5 min and a final extension cycle at 72°C for 15 min. The MUC1 primers produced a 288-bp PCR product.

For CK19, the primers used were:

CK19 – 5′ primer
5′-TTATTGGCAGGTCAGGAGAAGAGCC-3′;
CK19 – 3′ primer
5′-AGCTAACCATGCAGAACCTCAACGACCGC-3′.

As described by Datta et al (1994) the 3′ primer was designed to preclude transcription of pseudogenes.

The PCR conditions were 40 cycles of a single round carried out as described above to yield a 1097-bp PCR product. When two rounds of PCR were performed, nested primers were used as described by Datta et al (1994).

For CD44, the primers used were:

CD44(S) – 5′ primer
5′-AGTCACAGACCTGCCCAATGCCTTTG-3′;
CD44(S) – 3′ primer
5′-CACCTTCTTGACTCCCATGTGAGTGT-3′;
CD44(exons 8–11) – 5′ primer
5′-CTTGGATCCAACCACACCACGGGCTTTTGACCACA-3′;
CD44(exons 8–11) – 3′ primer
5′-CTTGGATCCTTCTTCCTGCTTGATGACCTCGTCCC-3′;
CD44(exons 12–14) – 5′ primer
5′-ATATGGACTCCAGTCATAGTACAACGCTTCAGC-3′;
CD44(exons 12–14) – 3′ primer
5′-CTGATAAGGAACGATTGACATTAGAGTTGGAAT-3′.

The PCR conditions were a first round of 20 cycles using primers to exons 4 and 16 of CD44, which produces a fragment of 486 bp from the standard form of CD44 mRNA found in leucocytes (Stamenkovic et al, 1989) in which the variant exons 6–15 have been spliced (Screaton et al, 1992). These primers also give rise to fragments that include the variant exons 6–14. For the second round of 30 cycles, 1–2 μl of first-round PCR product was used as a template in a 100 μl reaction containing either the exon 8 and 11 primers or exon 12 and 14 primers.

For all samples, the quality of RNA was routinely checked by running the standard form of CD44(S) as an internal control. 'No RT' controls for all RT-PCR reactions were run as above except that water was substituted for reverse transcriptase. Positive controls were RNA extracted from MCF-7 cell lines, and 'negative controls' contained all components of the RT-PCR reaction but no target RNA template.

### Gel electrophoresis of PCR products

RT-PCR products were analysed by 2% agarose gel electrophoresis and visualized through staining with ethidium bromide. A 100-bp DNA ladder or ΦX174 RF DNA/*Hae* III fragments (Gibco, Paisley, UK) were used as size markers.

### Southern blot analysis

PCR products were transferred from ethidium-stained agarose gels onto a nylon membrane (Hybond $N^+$, Amersham, UK) by overnight capillary transfer using 0.4 M sodium hydroxide (Sambrook et al, 1989). Filters were then placed in roller bottles (Becton Dickinson, Oxford, UK) and prehybridized at 65°C for

© Cancer Research Campaign 1998

4–6 h in 20 ml of prehybridization buffer containing 6 × SSC (0.9 M sodium chloride, 0.9 M sodium citrate), 10 × Denhardts (0.2% (w/v) each of Ficol, polyvinylpyrrolidone and bovine serum albumin) and 100 μl ml$^{-1}$ denatured sonicated salmon sperm DNA. The prehybridization buffer was then removed and 20 ml of hybridization fluid added containing 1 × 10$^6$ c.p.m. ml$^{-1}$ of $\gamma$-$^{32}$P-labelled oligonucleotide probe complementary to an internal sequence in the PCR product for MUCI, and hybridization was continued for a further 12–18 h. (For CK19 and CD44, the downstream primers were used as probes.) Oligonucleotide hybridization temperature was calculated from an empirical formula: $T_i$ = 4 (G or C) + 2 (A or T) – 5°C (Hames and Higgins, 1995). Filters were then washed at $T_i$ for 60 min in a buffer containing 6 × SSC, 0.1% sodium dodecyl sulphate (SDS) and for 5 min in the same buffer at $T_i$ + 5°C. At the completion of washing, filters were exposed to radiographic film (Kodak XAR-2 or equivalent) in cassettes with intensifying screen and exposed at –70°C for 12–36 h.

### RESULTS

The seeding experiments demonstrated a decreasing signal from MUC1 mRNA corresponding to a decreasing number of seeded tumour cells, reflecting an apparent sensitivity of one tumour cell per ml of blood. However, the unseeded blood sample in lane 1 showed a faint band, casting doubt on the specificity expression of MUC1 mRNA (Figure 1A). Further samples of normal peripheral blood showed variable expression of MUC1 mRNA in four of six volunteers (Figure 1B). A further experiment was performed using the standard form of CD44 [CD44(S)] mRNA as internal control. Similar background expression was demonstrated in peripheral blood samples from healthy human volunteers (Figure 1C). This variation in expression was not due to degradation of RNA in some specimens, as CD44(S) was equally represented in each (Figure 1D). In total, peripheral blood from 21 of 23 volunteers showed positive bands when assayed for MUC1 mRNA.

The results of the assay for variants of CD44 in peripheral blood from ten healthy volunteers is depicted in Figure 2. The metastasis-associated variant (exons 8–11) mRNA is present in four of ten samples.

In contrast, CK19 mRNA was not detected in normal peripheral blood (Figure 3). However, under the experimental conditions we used, the seeding experiments demonstrated that it was impossible to detect tumour cells at concentrations less than 10$^4$ cells per 10 ml of blood, i.e. one tumour cell per 10$^4$ white cells (Figure 4). This failure of detection did not appear to be due to degradation of RNA, as CD44(S)mRNA was detected in all these samples by RT-PCR (Figures 3A and 4A). In order to increase the sensitivity of detection, we attempted to use nested primers as described by Datta et al (1994), but abandoned this in light of frequency of detection of CK19 mRNA in blood from healthy volunteers.

### DISCUSSION

We previously reported the results of a pilot study in which we concluded that DF3 antigen (MUC1) may be used as a specific marker for detecting circulating tumour cells in patients undergoing surgery for breast cancer. Under similar experimental conditions, we have demonstrated background expression of MUC1 in the peripheral blood of 21 of 23 healthy human female volunteers. In support of these findings, Hoon et al (1995) also detected

**Figure 1** Sensitivity and specificity of MUC1 mRNA. (**A**) Autoradiographic detection of MUC1 mRNA in blood samples from a healthy subject mixed with serial dilutions of MCF-7 cells after 30 cycles of PCR amplification and hybridization with $^{32}$P-labelled MUC1 oligonucleotide. Lanes 1–6: 0, 10, 10$^2$, 10$^3$, 10$^4$ and 10$^5$ MCF-7 cells per 10 ml of blood respectively; lane 7, MCF-7 mRNA. (**B**) Autoradiograph of Southern blot showing hybridization of $^{32}$P-labelled MUC1 oligonucleotide to 30 cycles of PCR product. It demonstrates 288-bp DNAs in the MCF7 mRNA (lane 8) and in four out of six normal peripheral blood samples (lanes 2–7). Lane 1, 'no RT' control. (**C**) Ethidium bromide-stained agarose gel of RT-PCR product from MUC1 mRNA in five other normal bloods (lanes 2–6). Lane 1, 'no RT' control; lane 7, MCF-7 mRNA. (**D**) Ethidium bromide-stained agarose gel of RT-PCR product for CD44(S) mRNA as internal control for the integrity of RNA of all samples of normal blood and MCF-7 mRNA in Figure 1C

MUC1 expression in seven of eight normal donor peripheral blood leucocytes and four normal lymph nodes.

Our results also raise serious doubts about the value of using CD44 (exons 8–11) mRNA to detect circulating tumour cells because of the frequency with which it is found in normal peripheral blood. It is not known whether the expression of these two markers in normal peripheral blood is due to a small amount of expression of genes in a proportion of peripheral blood cells, or due to the presence of a few epithelial cells in the blood of some individuals.

CK19 mRNA has been claimed to be a specific epithelial cell marker (Datta et al, 1994). However, the sensitivity of detection of

© Cancer Research Campaign 1998

**Figure 2** Detection of CD44 (exons 8–11) mRNA in normal blood. Ethidium bromide-stained agarose gel of RT-PCR product for CD44 mRNA showing expression of exons 8–11 in four out of ten samples of peripheral blood from healthy volunteers (lanes 4, 5, 8 and 10). The RT-PCR product of exons 12–14 is used here as internal control. Lane 11, MCF-7 mRNA; lane 12, 'no RT' control

**Figure 3** Specificity of CK19 mRNA. (**A**) Ethidium bromide-stained agarose gel showing 486-bp bands of CD44(S) as internal control for five blood samples (lanes 2–6) and MCF-7 cells (lane 7). (**B**) Ethidium bromide-stained agarose gel of RT-PCR product from CK19 mRNA. Lane 7, MCF-7 mRNA; lanes 2–6, normal blood samples showing no visible bands. (**C**) Southern blot showing hybridization of $^{32}$P-labelled CK19 oligonucleotide to 40 cycles of PCR product. This shows a 1069-bp band in the MCF-7 cells and confirms its absence in five normal blood samples. Lane 1, 'no RT' control

CK19 in a single round of amplification was limited to greater than $10^4$ cells per 10 ml of blood. Because of the frequency with which CK19 mRNA was detected after two amplification rounds in blood from healthy volunteers, we abandoned using the nested primers described by Datta et al (1994), which were designed to increase sensitivity. We also found increasing the number of cycles in a single amplification round above 40 cycles enhanced sensitivity but reduced specificity, as previously reported by Schoenfield et al (1994).

Several repetitions of this experiment produced the same results. The failure to detect lower concentrations of tumour cells

**Figure 4** Sensitivity of CK19 mRNA. (**A**) Ethidium bromide-stained agarose gel showing bands of CD44(S) as internal control for the integrity of RNA. (**B**) RT-PCR detection of CK19 mRNA in blood samples from a healthy subject mixed with serial dilutions of MCF-7 cells detected on ethidium bromide-stained agarose gel after 40 cycles of PCR amplification. (**C**) Southern blot and hybridization of $^{32}$P-labelled CK19 oligonucleotide to 40 cycles of PCR product of Figure 4B. Lane 1, 'no RT' control; lanes 2–8: 0, 10, $10^2$, $10^3$, $10^4$, $10^5$ and $10^6$ MCF-7 cells per 10 ml of blood respectively did not appear to be due to degradation of RNA, as undegraded CD44(S) mRNA was detected in these samples by RT-PCR. Our studies clearly demonstrate that, while RT-PCR has the potential to detect minute quantities of a specific RNA sequence against a background of a vast excess of other RNA, there are dangers in assuming that the technique can be used to detect minute numbers of specific cells because of the well-established phenomenon of illegitimate transcription, which results in very low levels but non-specific expression of irrelevant genes in many cell types (Chelly et al, 1989). Illegitimate transcription limits the usefulness of RT-PCR to detect another putative epithelial cell-specific mRNA (De Graaf et al, 1997).

The technique of immunobead–PCR may be a solution to the problem if combined with detection of an irrefutable genetic marker of a tumour, such as Ki-*ras* mutations (Hardingham et al, 1993). It may be applicable in those tumours that carry the mutated gene. Such a specific mutation is as yet unknown in breast cancer. Further work is essential to identify a suitable epithelial marker that has the required specificity and sensitivity to be used reliably in the detection of circulating tumour cells from solid tumours, and meticulous attention to methodology is required to overcome the technical problems with this method before its broader use in clinical practice can be recommended.

### REFERENCES

Abe M and Kufe D (1993) Characterisation of cis-acting element regulating transcription of the human DF3 breast carcinoma-associated antigen (MUC1) gene. *Proc Natl Acad Sci USA* **90**: 282–286

Brown DC, Purushotham AD, Birnie GD and George WD (1995) Detection of intraoperative tumour-cell dissemination in patients with breast-cancer by use of reverse transcription and polymerase chain reaction. *Surgery* **117**: 96–101

Burchill SA, Bradbury FM, Smith B, Lewis IJ and Selby P (1994) Neuroblastoma cell detection by reverse transcriptase-polymerase chain reaction (RT-PCR) for tyrosine hydroxylase mRNA. *Int J Cancer* **57**: 671–675

Burchill SA, Bradbury MF, Pittman K, Southgate J, Smith B and Selby P (1995) Detection of epithelial cancer cells in peripheral blood by reverse transcriptase-polymerase chain reaction. *Br J Cancer* **71**: 278–281

© Cancer Research Campaign 1998

Chelly J, Concordet JP, Kaplan JC and Kahn A (1989) Illegitimate transcription: transcription of any gene in any cell type. *Proc Natl Acad Sci USA* **86**: 2607–2621

Choy A and McCulloch P (1996) Induction of tumour cell shedding into effluent venous blood breast cancer surgery. *Br J Cancer* **73**: 79–82

Datta YH, Adams PT, Drobyski WR, Ethier SP, Terry VH and Roth MS (1994) Sensitive detection of occult breast cancer by the reverse transcriptase polymerase chain reaction. *J Clin Oncol* **12**: 475–482

de Graaf H, Maelandsmo GM, Ruud P, Forus A, Oyjord T, Fodstad O and Hovig E (1997) Ectopic expression of target genes may represent an inherent limitation of RT-PCR assays used for micrometastasis detection: studies on the epithelial glycoprotein gene EGP-2. *Int J Cancer* **72**: 191–196

Delatorre M, Heldin P and Bergh J (1995) Expression of the CD44 glycoprotein (lymphocyte-homing receptor) in untreated human breast-cancer and its relationship to prognostic markers. *Anticancer Res* **15**: 2791–2795

Diel IJ, Kaufmann M, Goerner R, Costa SD, Kaul S and Bastert G (1992) Detection of tumour cells in bone marrow of patients with primary breast cancer: a prognostic factor for distant metastasis. *J Clin Oncol* **10**: 1534–1539

Dilworth D and McCarrey J (1992) Single-step elimination of contaminating DNA prior to reverse transcriptase PCR. *PCR Method Appl* **1**: 279–282

Eschwege P, Dumas F, Blanchet P, Le Maire V, Benoit G, Jardin A, Lacour B and Loric S (1995) Haematogenous dissemination of prostatic epithelial cells during radical prostatectomy. *Lancet* **346**: 1528–1530

Feuer EJ, Wun WM, Boring CC, Flanders D, Timmel MJ and Tong T (1993) The lifetime risk of developing breast cancer. *J Natl Cancer Inst* **85**: 892–897

Fox SB, Fawcett J, Jackson DG, Collins I, Gatter KC, Harris AL, Gearing A and Simmons DL (1994) Normal human tissues, in addition to some tumours, express multiple different CD44 isoforms. *Cancer Res* **54**: 4539–4546

Frank JA, Ling A, Patronans NJ, Carrasquillo JA, Horvath K, Hickey AM and Dwyet AJ (1990) Detection of malignant bone tumors: MR imaging vs scintigraphy. *Am J Roentgenol* **155**: 1043–1048

Gross HJ, Verwer B, Houck D, Hoffman RA and Rectenwald D (1995) Model study detecting breast cancer cells in peripheral blood mononuclear cells at frequencies as low as 10(–7). *Proc Natl Acad Sci USA* **92**: 537–451

Gunthert U, Hofmann M, Rudy W, Reber S, Zoller M, Haussmann I, Matzku S, Wenzel A, Ponta H and Herlich P (1991) A new variant of glycoprotein CD44 confers metastatic potential to rat carcinoma cells. *Cell* **65**: 13–24

Hames BD and Higgins SJ (1995) *Gene Probe 1: a Practical Approach.* Oxford University Press: New York

Hardingham JE, Kotasek D, Farmer B, Butler N and Mi JX (1993) Immunobead-PCR: a technique for the detection of circulating tumour cells using immunomagnetic beads and the polymerase chain reaction. *Cancer Res* **53**: 3455–3458.

Hardingham JE, Kotasek D, Sage RE, Eaton MC, Pascoe VH and Dobrovic A (1995) Detection of circulating tumour cells in colorectal cancer by immunobead-PCR is a sensitive prognostic marker for relapse of disease. *Mol Med* **1**: 789–794

Ho SB, Niehans GA, Lyftogt C, Yan PS, Cherwitz DL, Gum ET, Dahiya R and Kim YS (1993) Heterogeneity of mucin gene expression in normal and neoplastic tissues. *Cancer Res* **53**: 641–651

Hofmann M, Rudy W, Zoller M, Tolg C, Ponta H, Herrlich P and Gunther U (1991) CD44 splice variant confer metastatic behaviour in rats: homologous sequences are expressed in human tumour cell lines. *Cancer Res* **51**: 5292–5297

Hoon D, Doi F, Giuliano A, Schmid P and Conrad A (1995) The detection of breast carcinoma micrometastases in axillary lymph nodes by means of reverse transcriptase–polymerase chain reaction. *Cancer* **76**: 533–535

Johnson PWM, Burchill SA and Selby PJ (1995) The molecular detection of circulating tumour cells. *Br J Cancer* **72**: 268–276

Matsumura Y and Tarin D (1992) Significance of CD44 gene products for cancer diagnosis and disease evaluation. *Lancet* **340**: 1053–1058

McCulloch P, Choy A and Martin L (1995) Association between tumour angiogenesis and tumour cell shedding into effluent venous blood during breast cancer surgery. *Lancet* **346**: 1334–1335

Moll R, Franke WW, Schiller DL and Geiger B (1982) The catalogue of human cytokeratins: patterns of expression in normal epithelia, tumours and cultured cells. *Cell* **31**: 11–24

Papadimitriou JT, Berdichevsky F, D'Souza B and Burchell J (1993) Human models of breast cancer. *Cancer Surv* **16**: 59–69

Sambrook J, Fritsch EF and Maniatis T (1989) *Molecular Cloning: a Laboratory Manual.* Cold Spring Harbor Laboratory Press: Cold Spring Harbor

Schoenfeld A, Luqmani Y, Smith D, O'Reily S, Shousha S, Sinnett HD and Coombes C (1994) Detection of breast cancer micrometastases in axillary lymph nodes by using polymerase chain reaction. *Cancer Res* **54**: 2986–2990

Screaton GR, Bell MV, Jackson DG, Cornelis FB, Gerth U and Bell JI (1992) Genomic structure of DNA coding the lymphocyte homing receptor CD44 reveals at least 12 alternatively spliced exons. *Proc Natl Acad Sci USA* **89**: 12160–12164

Smith B, Selby P, Southgate J, Pittman K, Bradley C and Blair G (1991) Detection of melanoma cells in peripheral blood by means of reverse transcriptase and polymerase chain reaction. *Lancet* **338**: 1227–1229

Stamenkovic I, Amiot M, Pseudo J and Seed B (1989) A lymphocyte molecule implicated in lymph node homing is a member of the cartilage link protein family. *Cell* **56**: 1057–1062

Steward CG, Goulden NJ, Katz F, Baines D, Martin PG, Langlands K, Potter MN, Chessells JM and Oakhill A (1994) A polmerase chain reaction study of the stability of Ig heavy-chain and T-cell receptor delta-gene rearrangements between presentation of childhood B-lineage acute lymphoblastic leukemia. *Blood* **83**: 1355–1362

Yamada M, Wassermann R, Lange B, Reichard BA, Womer RB and Rovera G (1990) Minimal residual disease in childhood B-lineage lymphoblastic leukemia: persistence of leukemic cells during the first 18 months of treatment. *N Engl J Med* **323**: 448–455

© Cancer Research Campaign 1998